# TMC function, dysfunction, and restoration in mouse vestibular organs

**DOI:** 10.3389/fneur.2024.1356614

**Published:** 2024-04-04

**Authors:** Evan M. Ratzan, John Lee, Margot A. Madison, Hong Zhu, Wu Zhou, Gwenaëlle S. G. Géléoc, Jeffrey R. Holt

**Affiliations:** ^1^Department of Otolaryngology, F.M. Kirby Neurobiology Center, Boston Children’s Hospital and Harvard Medical School, Boston, MA, United States; ^2^Department of Otolaryngology - Head and Neck Surgery, University of Mississippi Medical Center, Jackson, MS, United States; ^3^Department of Neurology, Boston Children’s Hospital and Harvard Medical School, Boston, MA, United States

**Keywords:** vestibular, hair cell, utricle, saccule, semicircular canal, TMC1, TMC2

## Abstract

*Tmc1* and *Tmc2* are essential pore-forming subunits of mechanosensory transduction channels localized to the tips of stereovilli in auditory and vestibular hair cells of the inner ear. To investigate expression and function of *Tmc1* and *Tmc2* in vestibular organs, we used quantitative polymerase chain reaction (qPCR), fluorescence in situ hybridization – hairpin chain reaction (FISH-HCR), immunostaining, FM1-43 uptake and we measured vestibular evoked potentials (VsEPs) and vestibular ocular reflexes (VORs). We found that *Tmc1* and *Tmc2* showed dynamic developmental changes, differences in regional expression patterns, and overall expression levels which differed between the utricle and saccule. These underlying changes contributed to unanticipated phenotypic loss of VsEPs and VORs in *Tmc1* KO mice. In contrast, *Tmc2* KO mice retained VsEPs despite the loss of the calcium buffering protein calretinin, a characteristic biomarker of mature striolar calyx-only afferents. Lastly, we found that neonatal *Tmc1* gene replacement therapy is sufficient to restore VsEP in *Tmc1* KO mice for up to six months post-injection.

## Introduction

Inner ear dysfunction is a common disorder that affects at least 430 million people globally with most disabling cases involving a genetic component (1–3). One debilitating symptom of inner ear dysfunction is loss of balance, which affects an estimated 14.8% of adults in the U.S. annually (4). The sense of balance depends in part on mechanically sensitive hair cells that line the sensory epithelia of the inner ear’s vestibular organs, including the utricle, saccule, and three semicircular canals. Genetic mutations resulting in balance dysfunction often perturb the function of vestibular hair cells, which makes them potential targets for inner ear gene therapy.

Vestibular hair cells within the utricle and saccule sensory epithelia (collectively known as the vestibular maculas) detect linear head movements and gravity, while hair cells within the sensory epithelia (cristas) of the three semicircular canals detect rotational head movements. In all five vestibular organs, hair cells transduce mechanical movements into electrochemical signals that are relayed to the central nervous system. There are two types of vestibular hair cells, type I and type II, and failure of vestibular hair cells to transduce and transmit mechanical signals results in dizziness, imbalance, nausea, and other symptoms that can render patients severely disabled, particularly in old age. As a result, clarifying when and where the essential mechanosensory transduction components are expressed will be essential for optimizing genetic diagnostics and gene therapy interventions for vestibular hypofunction.

Transduction of vestibular signals depends on the *Tmc1* and *Tmc2* genes, which encode mechanosensitive ion channels found in stereovilli (aka stereocilia) at the apical surface of hair cells (5, 6). Although hearing loss associated with *TMC1* mutations is well documented in humans, there is limited evidence for vestibular dysfunction in patients lacking functional *TMC1* and no evidence for hearing or balance dysfunction due to mutations in human *TMC2*. Mice lacking the *Tmc1* gene are profoundly deaf and lack mechanosensory transduction in mature auditory hair cells (5, 6). Double knockout mice lacking *Tmc1* and *Tmc2* are profoundly deaf, have severe circling behavior, imbalance, and lack sensory transduction in auditory and vestibular hair cells at all developmental stages. While the data suggest that TMCs contribute to vestibular function in immature mice, how and when TMC1 and TMC2 contribute to vestibular function in adult mice remains unclear. Furthermore, regional contributions of TMC1 and TMC2 in vestibular organs, including expression in type I and type II hair cells, are poorly understood.

Recent work shows neonatal gene replacement therapy with *Tmc2* can restore some vestibular function in *Tmc1*/*2* double knockout mice (*Tmc1*/*2* DKO) (7). Likewise, *Tmc1* gene replacement restores hearing in *Tmc1* mutant mice (8). However, it is unknown whether *Tmc1* KO mice exhibit vestibular dysfunction, and if so, whether *Tmc1* gene replacement is sufficient to restore vestibular function. Since recent evidence suggests that childhood balance disorder rates are much higher than previously estimated (9–11), early gene therapy treatment of congenital balance dysfunction merits further investigation. Although gene replacement therapy can potentially ameliorate vestibular hypofunction symptoms, more work is needed to identify the optimal conditions for replacing components of the mechanosensory transduction complex.

A challenge facing *Tmc1* gene therapy in vestibular hair cells has been identifying when and where *Tmc1* and *Tmc2* expression arises. Previous evaluation showed that *Tmc1* is not expressed equally across hair cells in the vestibular maculas. X-gal staining of P28 transgenic mice with a *LacZ* reporter gene driven by the endogenous *Tmc1* or *Tmc2* promoters revealed a predominantly extrastriolar expression profile for *Tmc1*. *Tmc2* X-gal staining was not detected (5). Profiling of neonatal vestibular hair cells through qPCR, scRNA-seq, and single-cell proteomic analyses of chicken utricle hair cells showed *Tmc1* expression clustered in extrastriola hair cells, whereas *Tmc2* was found predominantly in striolar hair cells (12, 13). Additionally, in zebrafish, homologs *tmc1*, *tmc2a*, and *tmc2b* assemble in different hair cell subtypes with distinct morphologies (14–16). Taken together, these results support the hypothesis that there may be regional differences in *Tmc1* expression in mice, or unique expression based on hair cell type. Cellular and/or regional differences in *Tmc1* expression in the mouse and human vestibular maculas may have relevant functional implications for vestibular signaling. Furthermore, characterization of expression differences in mammalian *Tmc1* and *Tmc2* will be important for guiding the location and timing of *Tmc1* gene replacement therapy in the inner ear.

## Methods

### qPCR of *Tmc1* and *Tmc2*

Utricles and saccules were collected separately from five WT mice at postnatal time points: P2, P14, P28, P60. RNA extraction and purification utilized the RNeasy Mini Kit (Qiagen). Total RNA was measured by spectrophotometer (Nanodrop, ND100, Thermo Fisher Scientific) and reverse transcribed to cDNA using iScript cDNA Synthesis Kit (Bio-Rad). Quantitative PCR reactions were then performed with the TaqMan Gene Expression Assays with intron spanning *Tmc1* (5′-CATCTGCAGCCAACTTTGGTGTGT-3′ and 5′-AGAGGTAGCCGGAAATTCAGCCAT-3′) and *Tmc2* (5′-AGATCTTTGCGTTCCTTGCCAACC-3′ and 5′-GATCTTCTTTCGCAGCTGGGCATT-3′). TaqMan probes were labeled with FAM reporter dye (Applied Biosystems). Cycle threshold (Ct) values of triplicate reactions were measured for each sample. *Tmc1* and *Tmc2* plasmid DNA of known concentrations and lengths (1:10, 1:100, 1:1000, 1:10000, 1:100000, 1:1000000) were used to validate *Tmc1* and *Tmc2* primer efficiency and to estimate particle number across all time points.

### FISH-HCR

Ear capsules were removed and fixed in 1.5 mL 4% PFA/PBS under RNAse-free conditions, rocking at 4°C. Fixative was replaced with 100% MeOH and stored at −20°C until further use. Tissues were dissected in MeOH and then rehydrated through a graded methanol (25, 50, 75%) nuclease-free PBS series @ room temperature (RT). Tissues were washed in nuclease free PBS for 10 min at 25°C in 150 μL, then immersed ears in 150 μL of 100 μg/mL Proteinase K solution and incubated for 25 min @RT on a nutator. Tissues were post-fixed with 150 μL 4% PFA for 20 min @ RT on a nutator and washed in PBS/0.05% tween thrice in 150 μL for 5 min at 25°C. Tissues were then incubated in pre-hybridization buffer for 30 min at 37°C followed by incubation in 200 μL of probe hybridization buffer for 5 min. Finally, a pre-hybridization solution was prepared by adding 3.2 pmol of the desired probe, 16 nm in working solution into 200 μL of solution and embryos were incubated overnight at 37°C on a nutator in a pre-cleaned container which retains moisture.

For assessment of FISH following AAV injection at P1, mice with variable injection efficiency and VsEP thresholds were collected by P60 by the same process described above. Proteinase K concentration was increased to 125 μg/mL working solution and FISH was conducted otherwise similarly. Maculas were imaged at 7 μm depth with a range defined by the apex of phalloidin stained stereovilli containing *Tmc1* puncta and basally to by *Gfi1* signal. Striolar and extrastriolar utricular regions were defined by the presence or absence of *Ocm* signal, and by the distance from the lateral edge of the sensory domain. ROIs were restricted to hair cells expressing both *Gfi1* and phalloidin signals, and all *Tmc1* signal was normalized to the amount of *Gfi1* present in each tissue.

### FM1-43 labeling

P3 vestibular tissue was rapidly dissected following decapitation and placed under tungsten pins affixed to a glass coverslip using Sylgard. Tissue was submerged into 5 mL culture media containing 5 μM FM1-43 dye for 10–30 s with gentle agitation, transferred to a new container of 5 mL of culture media, washed three times, and placed in a slide holder for imaging. P60 mice were injected intraperitoneally with 5 mg/g mouse weight of FM1-43/FX fixable form as previously described (17). Tissue was collected after ~24 h and fixed for 1 h at 25°C in 4% PFA/PBS protected from light. Tissue was then transferred into 120 mM EDTA pH 7.4 for 48–72 h protected from light. Finally, tissue was transferred to PBS, dissected, and incubated in 1:1000 Phalloidin 405 Invitrogen Cat#A30104, Lot# 2403691.

### Immunohistochemistry

All tissue was fixed in 4% PFA/PBS for 1 h at room temperature before being removed and replaced with PBS for storage at 4°C. Tissue was later dissected in PBS and freeze/thaw permeabilized in 30% sucrose for up to 30 min on dry ice. Tissue was then blocked and permeabilized further using 5% donkey serum (DS), 1% bovine serum albumin (BSA), and 0.3% triton X-100 in PBS for 30 min. Solution was then removed, and tissue was rinsed three times with PBS/0.05% tween 20. Primary antibodies were prepared in a solution of 5% DS, 1% BSA, 0.3% triton in PBS. The following antibodies were used: Mouse anti-CtBP2 BD Transduction Labs Cat#612044 (1:200), Mouse anti βIII tubulin (Tuj1), Biolegend Cat #801202 Lot#B249869 (1:200), Swant Rabbit anti-Oncomodulin (1:200) Cat#OMG4, Rabbit anti-Myo7a Proteus Biosciences Cat#6790 (1:200), Guinea-pig anti-calretinin Swant Cat# CRgp7 (1:200). Invitrogen Phalloidin 405 (1:1000), Cat#A30104, Lot#2403691, Goat anti-mouse IgG1(y1) 2 mg/mL Alexa 633, Thermo Fisher Cat#A21126, Lot#73B1-1 (1:1000), Goat anti-Rabbit IgG Alexa 488 2 mg/mL, Thermo Fisher Cat#A11008, Lot#2179202 (1:1000), Donkey anti-Mouse Alexa 488 Thermo Fisher Ref#A21202, Lot#45966A (1:1000), Donkey anti-Guinea Pig Alexa 647 Jackson Labs Ref#A30104, Lot#2403691 (1:1000).

### Quantification of hair cells

Myo7a labeled tissues were imaged at 63X and tiled across all regions. Absolute total hair cell counts were completed using the FIJI (ImageJ) cell counter plugin across utricles, saccules, and cristas by three independent analysts (ER, JL, and MM). Quantification compared means ± standard error of the mean (SEM).

### Quantification of synapses

Quantification of CtBP2+ puncta was done with images of two striolar regions and two extrastriolar regions acquired from each utricle and saccule using a 63 × 1.4 NA oil objective lens (Carl Zeiss, *z* step = 0.36 μm, scaling per pixel: 0.068 μm × 0.068 μm; 2.5× zoom). Approximate striolar and extrastriolar regions were identified using calretinin-staining, which labels striolar calyxes and extrastriolar type II hair cells. For calretinin-positive calyx counts, confocal *z*-stacks of the entire striolar region were acquired from each utricle and saccule using the 63× oil objective (Carl Zeiss, *z* step = 0.36 μm, scaling per pixel: 0.099 μm × 0.099 μm; 0.5× zoom). CtBP2(+) puncta and calretinin(+) calyx counts were completed in Imaris Software using 3D projections to assess puncta via the “Spots” tool for each *z*-stack. Puncta counts were divided by the hair cell number to calculate the average number of CtBP2+ puncta/HC. For calretinin-positive calyx counts, all confocal *z*-stacks of a given utricle or saccule’s striolar region were aligned into a single image using the Zen Connect feature. All image quantifications were assessed in both males and females with no sex differences identified, and as a result, data were binned together accordingly.

### Utricle injection of AAVs

Neonatal mice (P1) were anesthetized via a hypothermic ice bath for 5 min after applying topical lidocaine cream anesthetic Cat# 70512-030. Incision and puncture of the ear capsule were done under sterile conditions. Titers of 1.54 × 10^13^ gc/mL of AAV9-PHP.B-*CMV*-*Tmc1ex1*-*WPRE*, and 6.13 × 10^13^ gc/mL of the AAV9-PHP.B-*CMV*-*eGFP*-*WPRE* were injected into the left utricle at volumes of 1.2 μL per mouse. Injections were done in a small fenestration from a 30G needle (Lot#1253645) using a pulled glass capillary pipette with slow volumetric displacement from a 1 mL syringe pushing through a latex tube. Following injection, the incision site was sutured closed via surgeon’s knot and carefully trimmed. Mice recovered on a heating pad before being returned to the home cage as described previously (18).

### VsEPs

All live experiments were conducted following IACUC approval protocol #00001240. Mice were anesthetized with ketamine/xylazine stock injected at a working concentration of 125 mg/kg mouse body weight. After 6–10 min, mice were tested for responses to foot press. A 23-gauge needle was used to pierce the neck skin behind the nuchal crest to thread 5 cm of stripped stainless-steel fiber (A-M Systems, Cat. 791500) and tie a secure loop. The other end was alligator clipped to a reference electrode. When fully anesthetized, mice were placed on their backs with ground electrode placed near left hip bone and head was secured with padded spring clip before placing the recording electrode inferior to the pinna at an angle to prevent movement during acquisition. All cables were taped down flat, and animal body temperatures were monitored using a rectal thermometer. Calibration was done for each mouse head size to ascertain a sensitivity reading ranging from −10 to −14 dB re: g/ms. The mouse’s head was subsequently translated with shaker and monitored by accelerometer increasing in 3 dB step sizes from −22.4 to 1.6 dB re: g/ms. Thresholds were called based on the presence of at least one of three distinct peaks occurring at ~1, 2, and 3 ms as previously described (19).

### VORs

*Tmc1* KO (*n* = 10), *Tmc2* KO (*n* = 6), *Tmc1*/*2* DKO (*n* = 10), and WT C57BL6 mice (WT, *n* = 23) were assessed. VOR responses to sinusoidal head rotation (0.2–4 Hz) (rVORs) and translation (0.2–2 Hz) (tVORs) were recorded utilizing infrared eye tracking (20, 21). Briefly, steady-state VOR responses were measured with horizontal rotations delivered at 0.2, 0.5, 1, 2, and 4 Hz (60°/s peak velocity). Horizontal translations were delivered along 45° right from the nasal-occipital direction (i.e., the translation direction is perpendicular to the visual axis of the left eye) at 0.2, 0.5, 1, and 2 Hz with 0.1 g peak acceleration. A minimum of 30 cycles/50 trials were recorded per condition. Horizontal and vertical signals for eye position, head rotation, and translation, were processed via an analytical pipeline has been described previously (7). All mice assessed were aged P60-P90 under similar conditions.

## Results

### *Tmc1* expression increases with age, while *Tmc2* expression decreases

**Figure 1:**
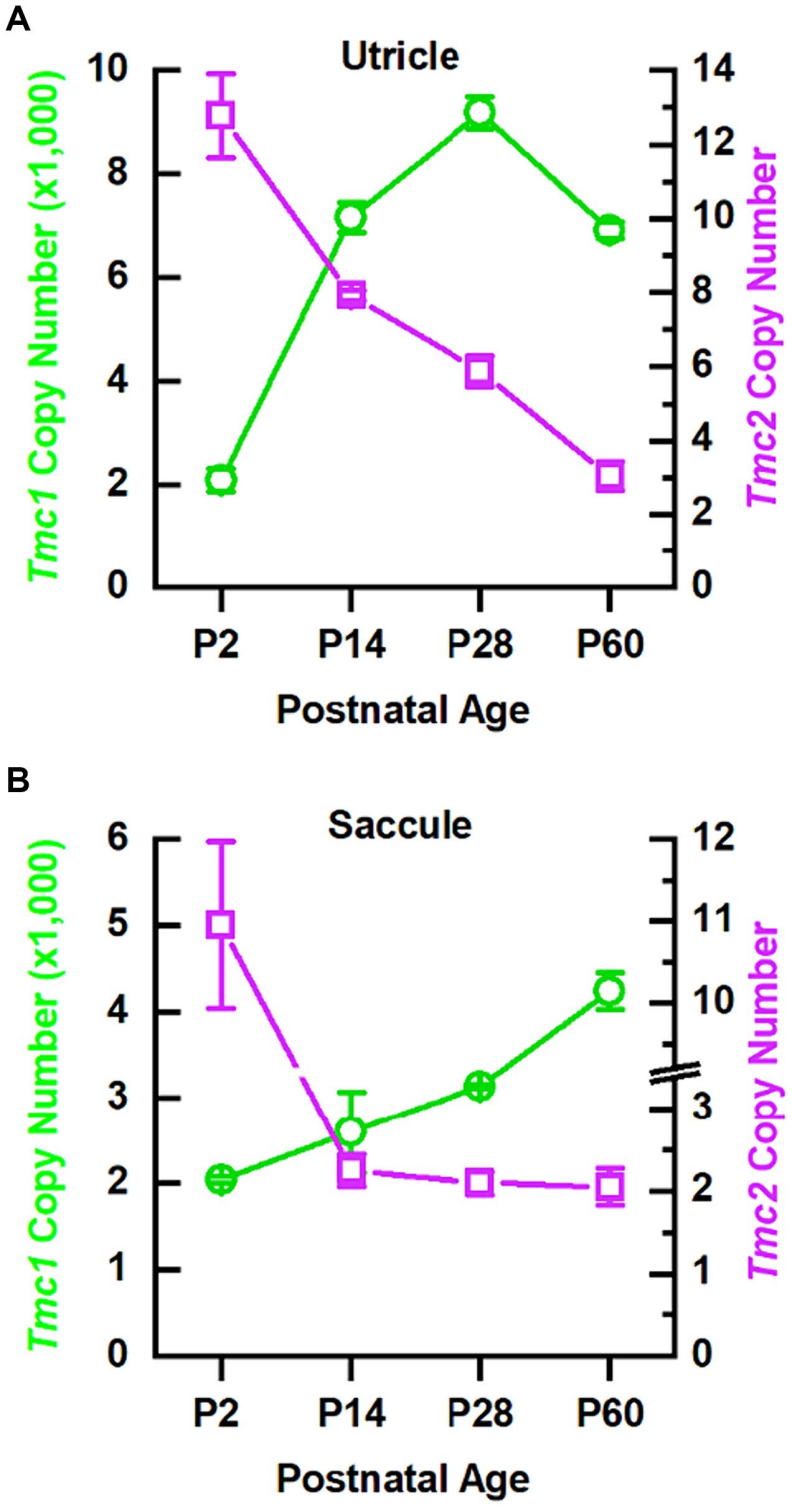
qPCR data indicate *Tmc1* levels increase while *Tmc2* levels decrease. **(A)** qPCR results for 10 utricles **(B)** and 10 saccules for each time point. *Tmc1* is indicated by green circles corresponding to the left *y*-axis, and *Tmc2* is indicated by magenta squares corresponding to the right *y*-axis. Data points represent mean ± SEM.

In the cochlea, there is a developmental switch from *Tmc2* to *Tmc1* expression during the first postnatal week, whereas, in the utricle, both *Tmc1* and *Tmc2* are expressed at least until P21 (5). Whether *Tmc* expression persists into adulthood in vestibular organs has not been clarified. To examine the relative abundance of *Tmc1* and *Tmc2* expression during postnatal development and into adulthood (P2, P14, P28, P60), utricles and saccules were dissected and analyzed separately via quantitative RT-PCR (qPCR). *Tmc2* expression levels were highest in both organs at P2 and declined through P60. *Tmc2* levels declined rapidly in the saccule and more gradually in the utricle (Figure 1). Interestingly, as *Tmc2* declined, levels of *Tmc1* expression rose, were ~1,000-fold higher than *Tmc2*, and increased as mice matured into adulthood and thus showed a similar but delayed trend compared to the cochlea (Figure 1). These data indicate that early neonatal vestibular development is characterized by a transitory wave of low levels of *Tmc2* expression that dissipates during adulthood, while *Tmc1* expression steadily rises into adulthood.

### *Tmc1* expression is reduced in the striola of neonatal mice

**Figure 2:**
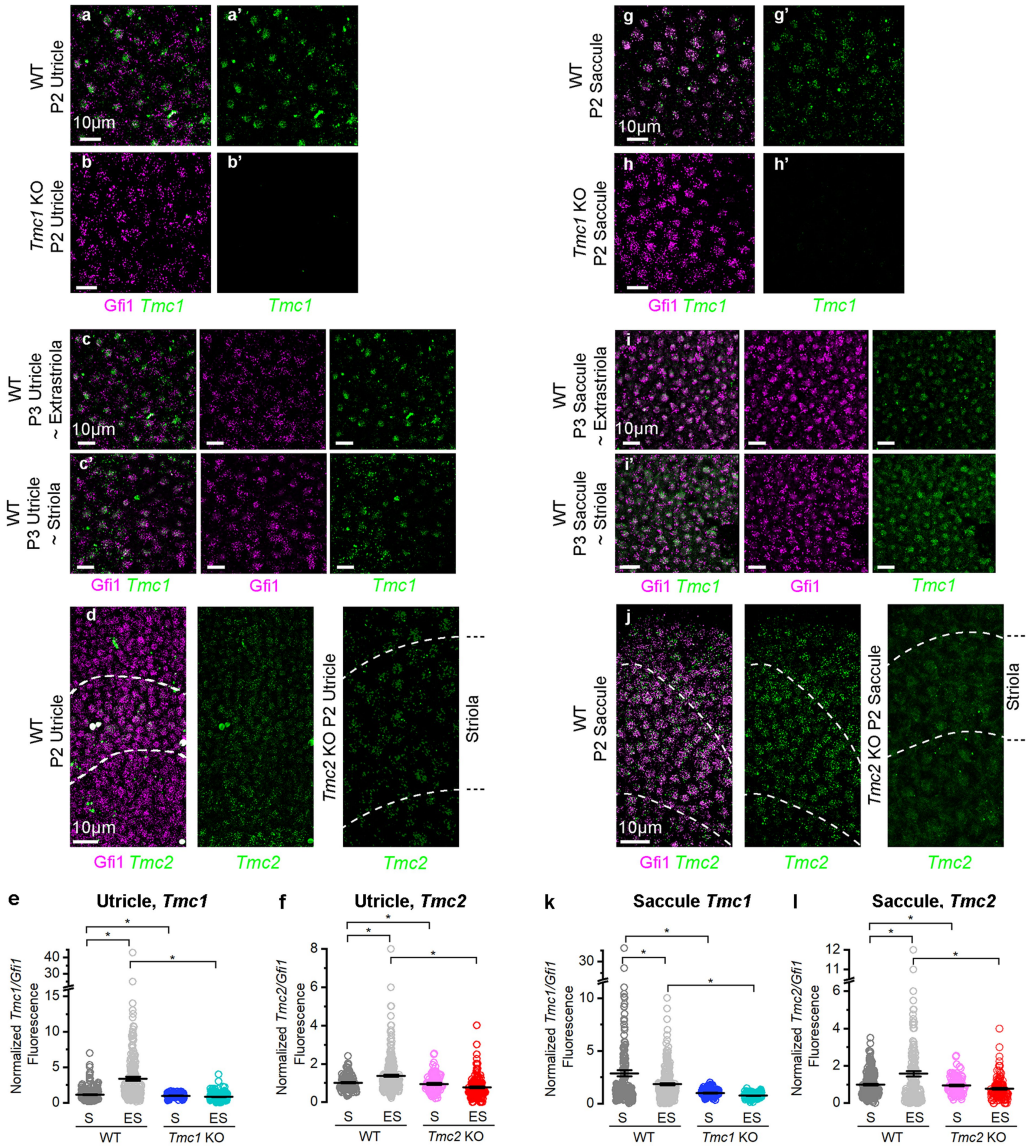
FISH-HCR analysis of *Tmc1* and *Tmc2* expression in vestibular maculas. **(A,A′)** *Gfi1* and *Tmc1* expression in wholemount P2 WT and **(B,B′)** *Tmc1* KO utricles. **(C,C′)** *Gfi1* and *Tmc1* expression in WT P3 utricle extrastriolar and striolar regions. **(D)** Striolar domain of the utricle defined by *Ocm* (dashed lines) and hair cells by *Gfi1*. *Tmc2* expression was seen throughout the sensory epithelia. **(E)** Quantification of *Tmc1* expression normalized to *Gfi1* in the extrastriolar versus striolar domain of utricle. **(F)** Quantification of normalized *Tmc2* expression in the extrastriolar versus striolar domain of the utricle. **(G,G′)** *Gfi1* and *Tmc1* expression in wholemount P2 WT and **(H,H′)** *Tmc1* KO saccules. **(I,I′)** *Gfi1* and *Tmc1* expression WT P3 saccule extrastriolar and striolar regions. **(J)** Striolar domain of the saccule defined by *Ocm* (dashed lines) and hair cells by *Gfi1*. *Tmc2* expression was seen throughout the sensory epithelia. **(K)** Quantification of *Tmc1* expression normalized to *Gfi1* in the extrastriolar versus striolar domain of saccule. **(L)** Quantification of normalized *Tmc2* expression in the extrastriolar versus striolar domain of the saccule. Individual data points and mean ± SEM are shown for panels **E,F,K,L**. Horizontal bars and stars (*) indicate statistical significance, *p* < 0.05. Scale bars = 10 μm in all images.

At neonatal stages, *Tmc1* expression is predominant in the extrastriola, and the same pattern was evident at P28 (5). To further investigate the regional expression patterns of *Tmc1* and *Tmc2*, we used fluorescent *in situ* hybridization hairpin chain reaction (FISH-HCR) to examine utricular *Tmc1* and *Tmc2* mRNA at P3. *Tmc1* and *Tmc2* mRNA signals were absent from *Tmc1* KO and *Tmc2* KO negative controls, respectively (Figures 2B,D,H,J). In WT utricles, *Tmc1* transcripts co-localized with *Gfi1* signals in vestibular hair cells (Figures 2A,C,G,I). To quantify regional expression of *Tmc1*, we estimated the number of HCR puncta within hair cells, defined by phalloidin and *Gfi1* regions of interest (ROIs), as previously described (22, 23). *Tmc1* expression was normalized to *Gfi1* signal per hair cell ROI. *Tmc1* mRNA was significantly reduced in the striola versus lateral extrastriola of the utricle (striolar mean ± SEM: 1.19 ± 0.33 vs. extrastriolar: 3.37 ± 0.33, *n* = 3, *p* = 0.00002) (Figures 2C′,E). Notably, *Tmc2* utricle expression was substantially lower than *Tmc1* and also showed slightly elevated extrastriolar expression (Figures 2D,F), which was quantifiably different (striolar mean ± SEM: 1.02 ± 0.08 vs. extrastriolar: 1.38 ± 0.08, *n* = 3, *p* = 0.00002).

Subsequently, we sought to address whether Tmc1 and Tmc2 mRNA levels varied in different regions of the saccule (Figures 2G–L). Mean striolar hair cell fluorescence of *Tmc1* was higher than extrastriolar levels by FISH (striola: 2.87 ± 0.14; extrastriola:1.84 ± 0.14, *n* = 3, *p* = 0.0002) (Figure 2K). The striola of the saccule is bisected by the line of polarity reversal (LPR), where half the striolar hair cells express *Emx2*, a known enhancer of *Tmc1* (23, 24). In contrast, *Emx2* is notably absent from the striola of the utricle, which may contribute to the difference in *Tmc1* expression between striolas of otolith organs. Mean *Tmc2* expression levels were much lower in the saccule than *Tmc1*, and *Tmc2* was enriched in the extrastriolar region (striola: 0.99 ± 0.15; extrastriola: 1.58 ± 0.15, *n* = 3, *p* = 0.0002) (Figure 2L). Our data show that *Tmc1* expression was much higher than *Tmc2* in saccular hair cells but demonstrated a less prominent regional difference in the saccule than in the utricle (Figures 2K,I).

### Saccular hair cells depend more on *Tmc1* expression with age

**Figure 3:**
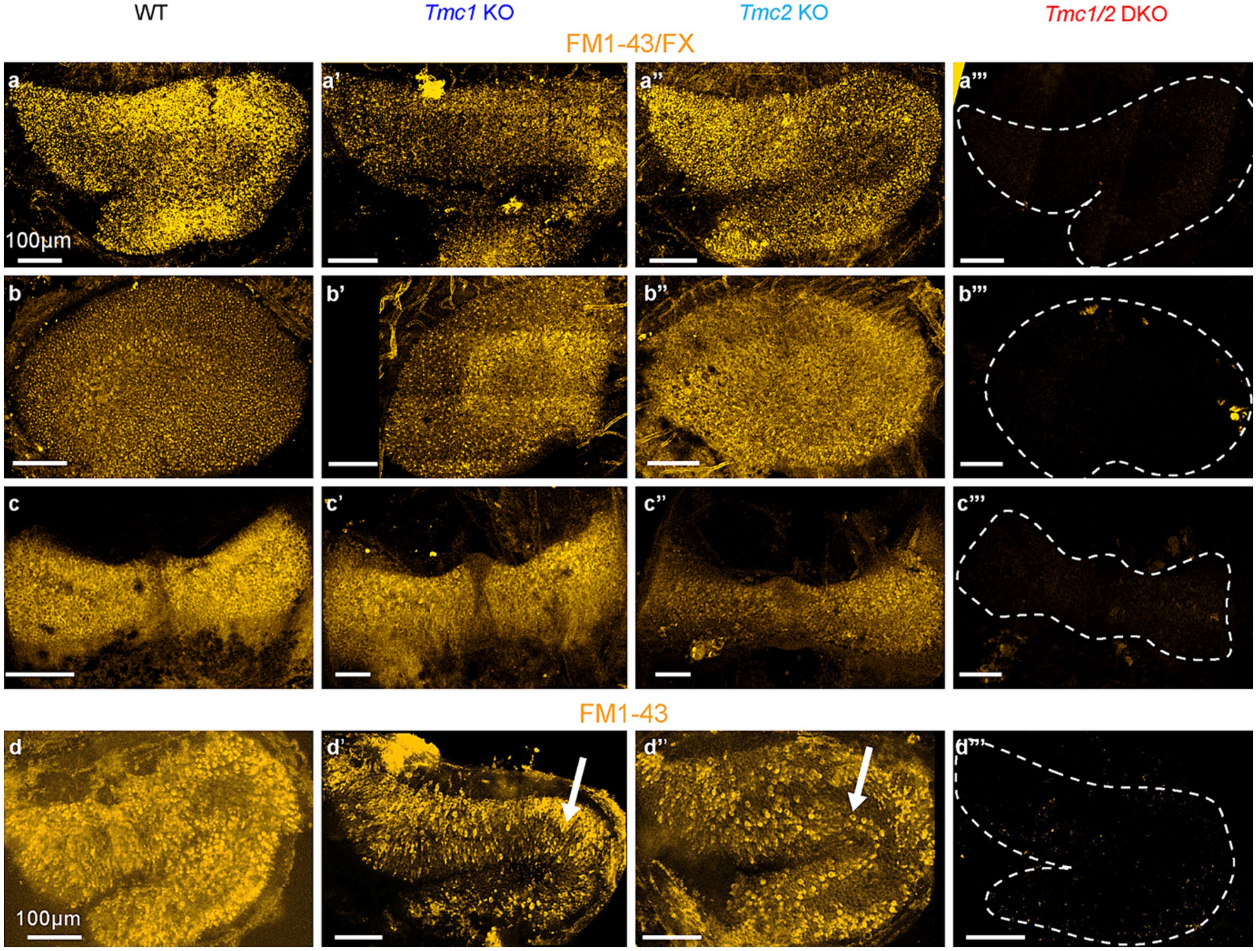
FM1-43 labeling of vestibular maculas from WT and *Tmc* mutant mice. **(A–A‴)** Saccules, **(B–B‴)** utricles, and **(C–C‴)** cristas collected from P60 mice IP-injected with 5 mg/kg of FM1-43/FX. Tissue was collected 24 h later, decalcified for 48 h, stained, and imaged. **(D–D‴)** P3 saccules labeled with bath-applied FM1-43 (not fixable). Scale bars = 100 μm.

The role of transient *Tmc2* expression in early neonatal vestibular development is unclear; however, one possible consequence of the decline in *Tmc2* expression in adulthood is an increased dependence on *Tmc1*, particularly in the saccule where *Tmc2* expression declines more rapidly. To test this hypothesis functionally, a fixable styryl dye (FM1-43/FX) was injected intraperitoneally at P60. Since FM1-43/FX enters hair cells through mechanosensory transduction channels (17), it is a useful proxy for visualizing functional hair cells. To compare dye uptake between genotypes, we dissected and imaged inner ear tissue from FM1-43/FX-injected mice. WT mice showed robust FM1-43/FX uptake in hair cells of the utricle, saccule, and semicircular canals (Figures 3A–C), while there was no uptake in any of the vestibular organs excised from *Tmc1*/*2* DKO mice (Figures 3A‴–C‴). At P60, *Tmc1* KO saccular hair cells appeared to take up less dye than WT and *Tmc2* KO counterparts (Figures 3A–A″). Additionally, at P60 the striola of *Tmc2* KO saccules was stained with dye similar to WT, despite a lack of staining at P3 (Figures 3D–D″). Interestingly, P60 *Tmc1* KO utricles resembled their P3 counterparts (5) but with even more diffuse dye labeling (Figures 3B–B″). These data suggest that the transitory wave of striolar *Tmc2* expression that occurs in neonates is less critical for dye uptake in saccular hair cells than the persistent expression of *Tmc1* in adulthood (P60).

### *Tmc1* KO and *Tmc2* KO mice do not lose vestibular hair cells

**Figure 4:**
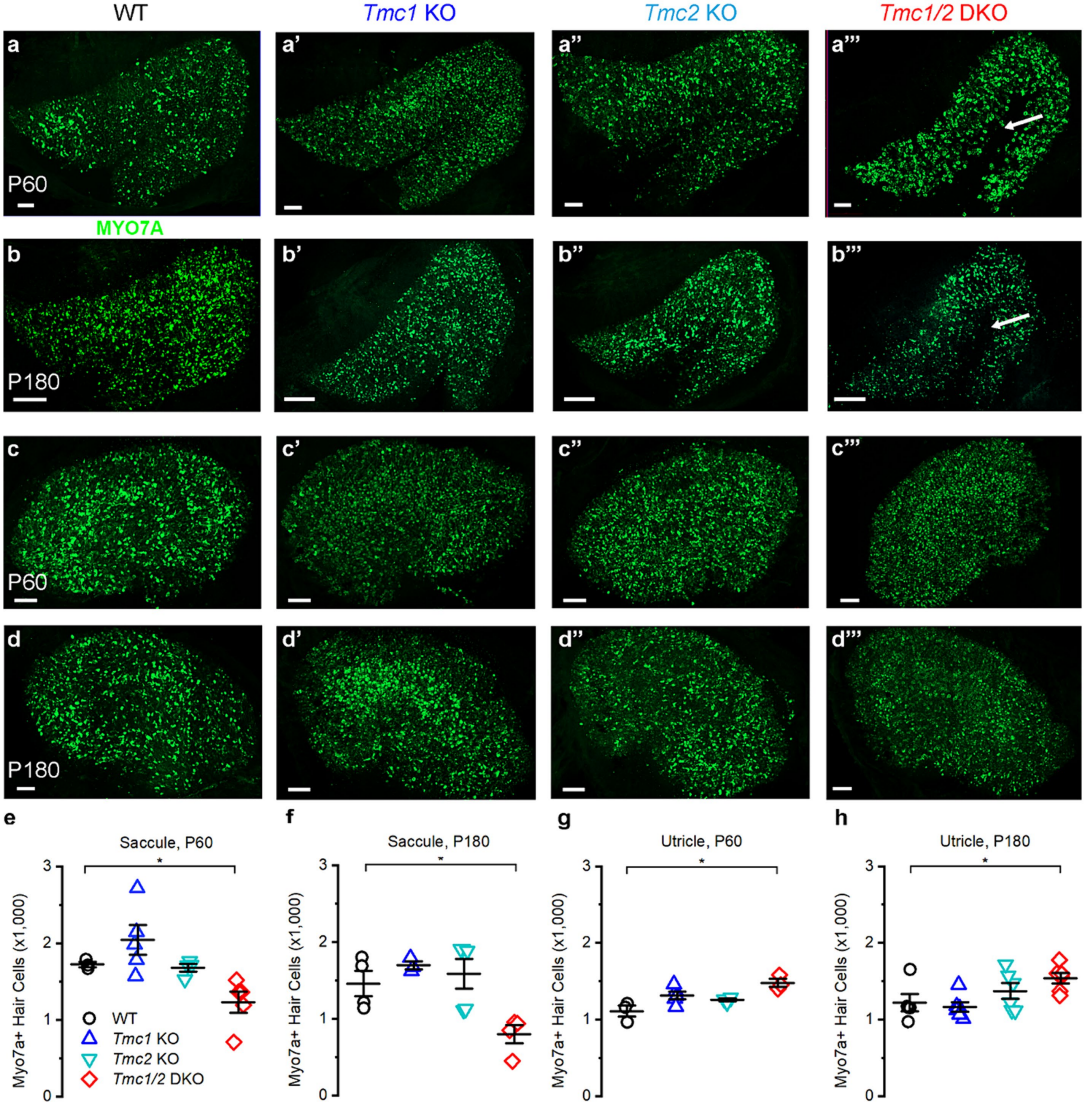
Myosin 7a staining in vestibular maculas of WT and *Tmc* mutant mice. **(A–A‴)** Confocal images of P60 saccules labeled with Myo7a from genotypes indicated above. **(B–B‴)** P180 saccules labeled with Myo7a antibody except where absent (arrow). **(C–C‴)** P60 utricles labeled with Myo7a antibody **(D–D‴)**. P180 utricles labeled with Myo7a antibody. **(E)** Quantification of Myo7a signal in P60 saccules across genotypes. **(F)** Quantification of Myo7a signal in P180 saccules across genotypes. **(G)** Quantification of Myo7a signal in P60 utricles. **(H)** Quantification of Myo7a signal in P180 utricles. Data points indicate number hair cells per tissue sample for each genotype with bars showing mean ± SEM in panels **E–H**. Horizontal bars and stars (*) indicate statistical significance, *p* < 0.05. Scale bars = 100 μm.

**Figure 5:**
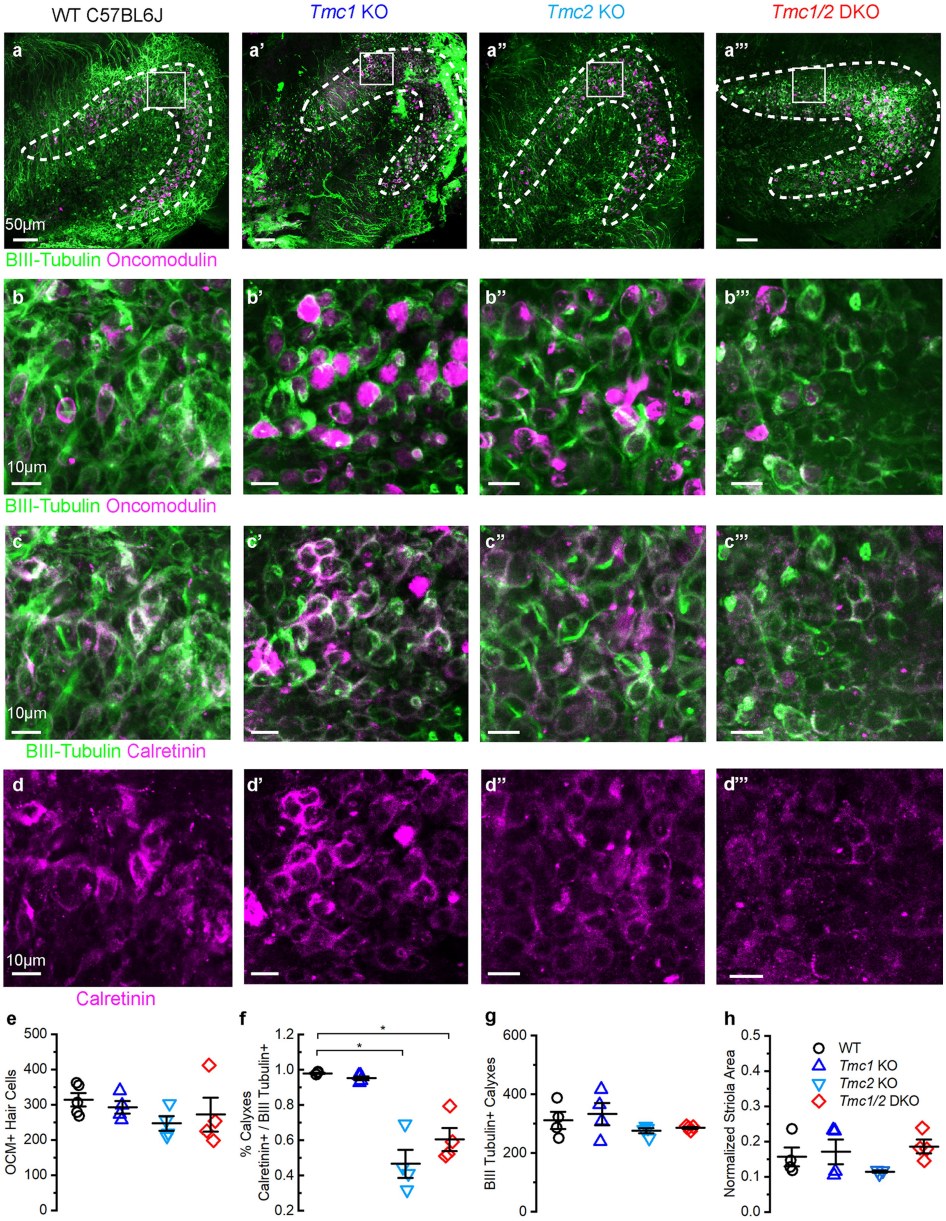
Immunostaining of P60 saccules from WT and *Tmc* mutant mice. **(A–A‴)** Saccular striola is defined by OCM labeling present in all genotypes (dashed lines) with higher magnification ROI indicated by square. **(B–B‴)** Type I striolar hair cells labeled by OCM (magenta) are contacted by calyxes labeled with βIII tubulin (green) in the striolar domain. **(C–C‴)** Calretinin expression labels striolar calyxes across all genotypes (magenta) and βIII tubulin expression labels calyxes across all genotypes (green). **(D–D‴)** Calretinin signal labels complex calyxes (magenta). **(E)** Quantification of total type I striolar hair cell numbers across genotypes at P60. **(F)** Quantification of the portion of striolar βIII tubulin(+) calyxes that also express calretinin across genotypes at P60. Horizontal bars and stars (*) indicate statistical significance, *p* < 0.05. **(G)** Quantification of the total number of βIII tubulin calyxes in the striola across genotypes at P60. **(H)** Quantification of striolar area based on OCM signal normalized to the total sensory domain labeled by phalloidin. Scale bars = 50 μm for **A**, and 10 μm for **B–D**. Data points indicate values for each genotype with bars showing mean ± SEM in panels **E–H**.

**Figure 6:**
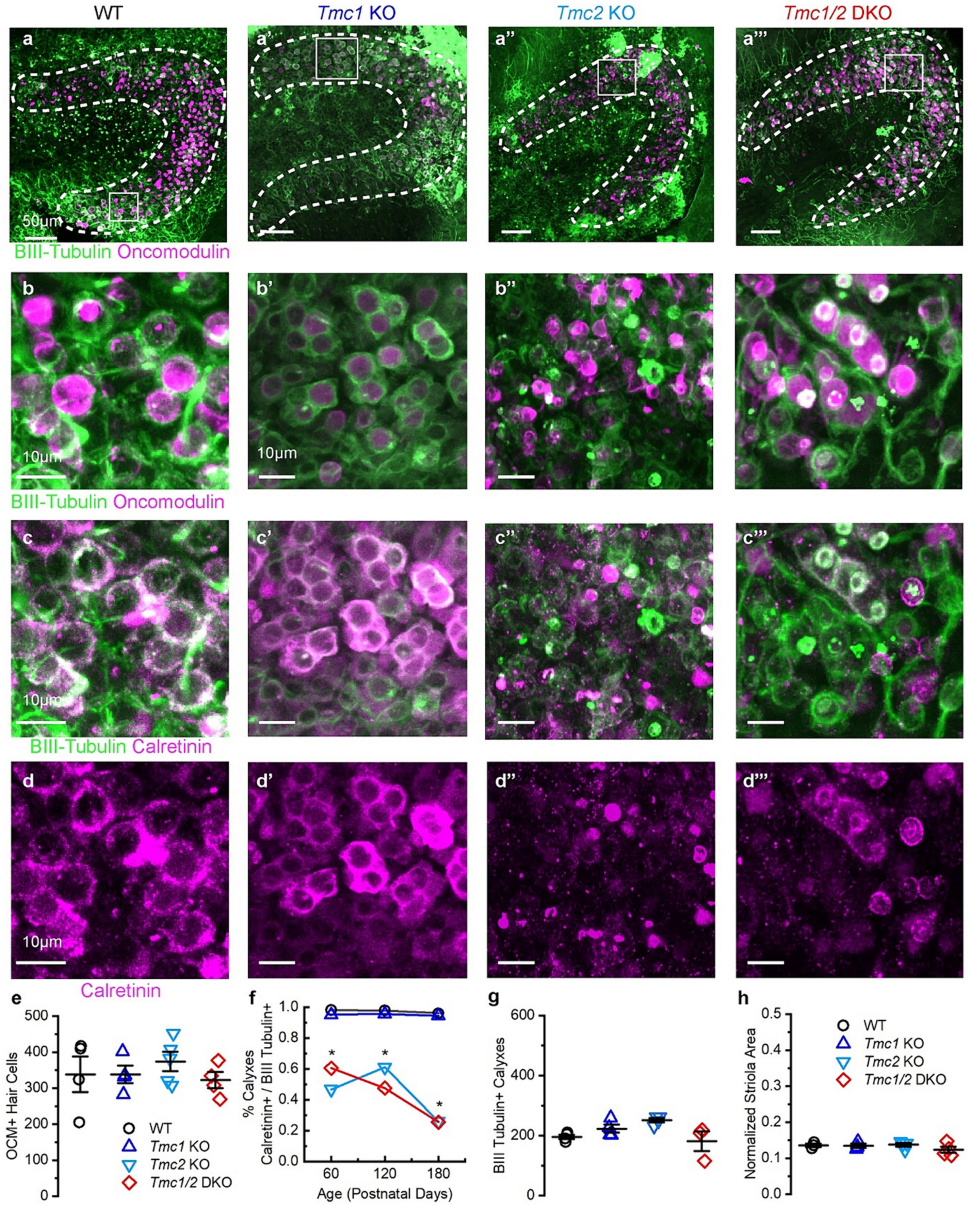
Immunostaining of P180 saccules from WT and *Tmc* mutant mice. **(A–A‴)** Saccule striola defined by OCM labeling present in all genotypes (dashed lines) with higher magnification ROI indicated by square. **(B–B‴)** Type I striolar hair cells are labeled by OCM (magenta) are contacted by calyxes labeled with βIII tubulin (green) in the striolar domain. **(C–C‴)** Calretinin expression labels striolar calyxes across all genotypes (magenta) and βIII tubulin expression labels calyxes across all genotypes (green). **(D–D‴)** Calretinin signal labels complex calyxes (magenta). **(E)** Quantification of total type I striolar hair cell numbers across genotypes at P180. **(F)** Quantification of the portion of striolar βIII tubulin(+) calyxes that also express calretinin across genotypes at P180. Stars (*) indicate statistical significance, *p* < 0.05. **(G)** Quantification of the total number of βIII tubulin calyxes in the striola across genotypes at P180. **(H)** Quantification of striolar area based on OCM signal normalized to the total sensory domain labeled by phalloidin. Scale bars = 50 μm for **A**, and 10 μm for **B–D**. Data points indicate values for each genotype with bars showing mean ± SEM in panels **E–H**.

In the cochlea, *Tmc1* loss of function mutations lead to cochlear hair cell death as early as 1 month of age (5, 8, 25, 26). To investigate the possibility of hair cell loss in the utricle and saccule of *Tmc* mutant mice, tissues were labeled with Myosin 7a (Figure 4). The total number of hair cells was visually counted for all genotypes in the saccule at P60 and P180. No significant loss was apparent in *Tmc1* KO, or *Tmc2* KO at P60 or P180 (Figures 4A–A″,B–B″) relative to WT. Despite the apparent necessity of *Tmc2* in the striola of the saccule during development, *Tmc2* KO, *Tmc1* KO, and *Tmc1*/*2* DKO saccules retained normal numbers of type I striolar hair cells based on OCM staining at both P60 and P180 (Figures 5E, 6E). However, *Tmc1*/*2* DKO saccules showed significant loss of hair cells, predominantly in the extrastriolar region internal to the line of polarity reversal (Figure 4B). In P60 *Tmc1*/*2* DKO saccules there were 1,414 ± 124 Myo7a-positive hair cells (*n* = 3, *p* = 0.04), while at P180 there were significantly fewer Myo7a-positive cells (661 ± 205, *n* = 3, *p* = 0.02). Thus, the combined loss of both genes in the *Tmc1*/*2* DKO mice led to significant loss of hair cells in the saccule but, surprisingly, not in the utricle. The mean number of Myo7a-positive cells in the utricle was unchanged across all genotypes except for *Tmc1*/*2* DKO utricles, which had a slight increase at P60 (1,478 ± 91, *n* = 3, *p* = 0.02) and P180 (1,392 ± 78, *n* = 5, *p* = 0.04).

### *Tmc* KO mice display no changes in striolar architecture of the adult utricle

Interestingly, the lack of FM1-43 uptake in P3 *Tmc2* KO maculas is predominantly in the striola where *Tmc1* expression is absent (Figure 3D″). Accordingly, we were curious to assess whether any changes occurred in the cellular architecture in the striolas of *Tmc2* KO mice. Oncomodulin (OCM) antibody labeling of type I striolar hair cells and calretinin antibody labeling of striolar calyx-only afferents were used to assess changes in the striolar region of WT, *Tmc1* KO, *Tmc2* KO, and *Tmc1*/2 DKO utricles (Supplementary Figure S1A) as previously described (27). The total number of calyx-bearing neuronal endings was evaluated using a βIII tubulin (Tuj1) antibody, which labels the striola domain (Supplementary Figures S1B,C). No difference in the total number of OCM-expressing type I striolar hair cells was apparent across genotypes (Supplementary Figure S1E). Calretinin expression in calyx-only afferents was quantified as a percentage of total calyx-containing βIII tubulin (+) cells within the striolar domain, the latter defined by OCM expression. No differences were identified in calretinin expression between genotypes (Supplementary Figures S1D,F). No differences in the total number of βIII tubulin (+) calyxes in the striola were identified across genotypes (Supplementary Figure S1G). In addition to the total number of neuronal endings and hair cells in the utricular striolas remaining unchanged, there was no change in the size of the striola region normalized to the total area of the sensory epithelia defined by phalloidin (Supplementary Figure S1H). The same analyses were completed at P180, showing no changes in the striola domain (Supplementary Figure S2). Taken together, these data indicate that loss of *Tmc1* and/or *Tmc2* does not alter the number of type I striolar hair cells or the number of calyceal afferent connections in the utricle.

### *Tmc2* KO and *Tmc1*/*2* DKO mice progressively lose calretinin expression in the saccule

While the saccules across genotypes showed no change in the total number of OCM(+) hair cells (Figures 5B,E), calretinin expression was diminished in afferent striolar neurons of *Tmc2* KO and *Tmc1*/*2* DKO saccules. No differences were observed in *Tmc1* KO (Figure 5F). By P60, 47.00 ± 0.08% of striolar calyx-only afferents expressed calretinin, compared to 98.00 ± 0.01% of WT (*n* = 4, *p* = 0.0007). Comparable loss of calretinin was observed in *Tmc1*/*2* DKO saccules where 60.00 ± 0.70% of striolar calyxes expressed calretinin (*n* = 4, *p* = 0.0012). Although statistically significant, *Tmc1* KO calretinin expression was reduced by just 2.70 ± 0.01%, suggesting that it plays less of an effect on striolar afferents than *Tmc2* (Figure 5F) (*n* = 4, *p* = 0.047). Despite a significant reduction in calretinin expression in *Tmc2* KO saccules, the total number of βIII tubulin(+) calyxes in the striola was unchanged, indicating otherwise normal innervation (Figure 5G). Likewise, the total area of the striola was also unchanged across genotypes in the saccule (Figure 5H). *Tmc2* appears to be uniquely important for expression of calretinin in striolar calyx-only afferents in the saccule, while *Tmc1* is less critical. Examination of P180 saccular striolas revealed that calretinin loss appears to be progressive, with only 28.00 ± 0.04% of *Tmc2* KO and 25.00 ± 0.03% of *Tmc1*/*2* DKO striolar calyxes having detectable calretinin by P180 (*n* = 4, *p* < 0.0005) (Figures 6C,D,F). Together with the lack of change in calretinin expression in the utricle, these results suggest that transient neonatal expression of *Tmc2* in the striola is more critical for the saccule.

### *Tmc1* and *Tmc2* KO mice show no changes in presynaptic ribbons

Since *Tmc1* and *Tmc2* expression affects synaptic development and maturation in the cochlea (28), we wondered whether regional expression differences of *Tmc1* and *Tmc2* may affect presynaptic ribbon distribution within striolar versus extrastriolar hair cells in vestibular macules. Ribbon counts from the approximate striolar and extrastriolar regions were conducted using CtBP2 and calretinin antibody labeling. The regional demarcations are approximate due to the loss of calretinin from *Tmc2* KOs and *Tmc1*/*2* DKOs in the saccule. Reduced numbers of CtBP2(+) puncta were observed predominantly in the extrastriola of *Tmc1*/*2* DKO saccules (Supplementary Figures S3C,D). To normalize ribbon counts to regional differences in hair cell densities and variable hair cell survival, the average number of ribbons per hair cell was calculated. Consistent with Myo7a staining, hair cell loss was evident in *Tmc1*/*2* DKO saccules by P180 (Supplementary Figures S3C,D). However, there were no significant differences in average ribbon counts across groups and time points (Supplementary Figures S3E,F). Loss of *Tmc1* or *Tmc2* does not appear to alter ribbon density in vestibular hair cells.

### *Tmc1* KO mice exhibit otolith dysfunction

**Figure 7:**
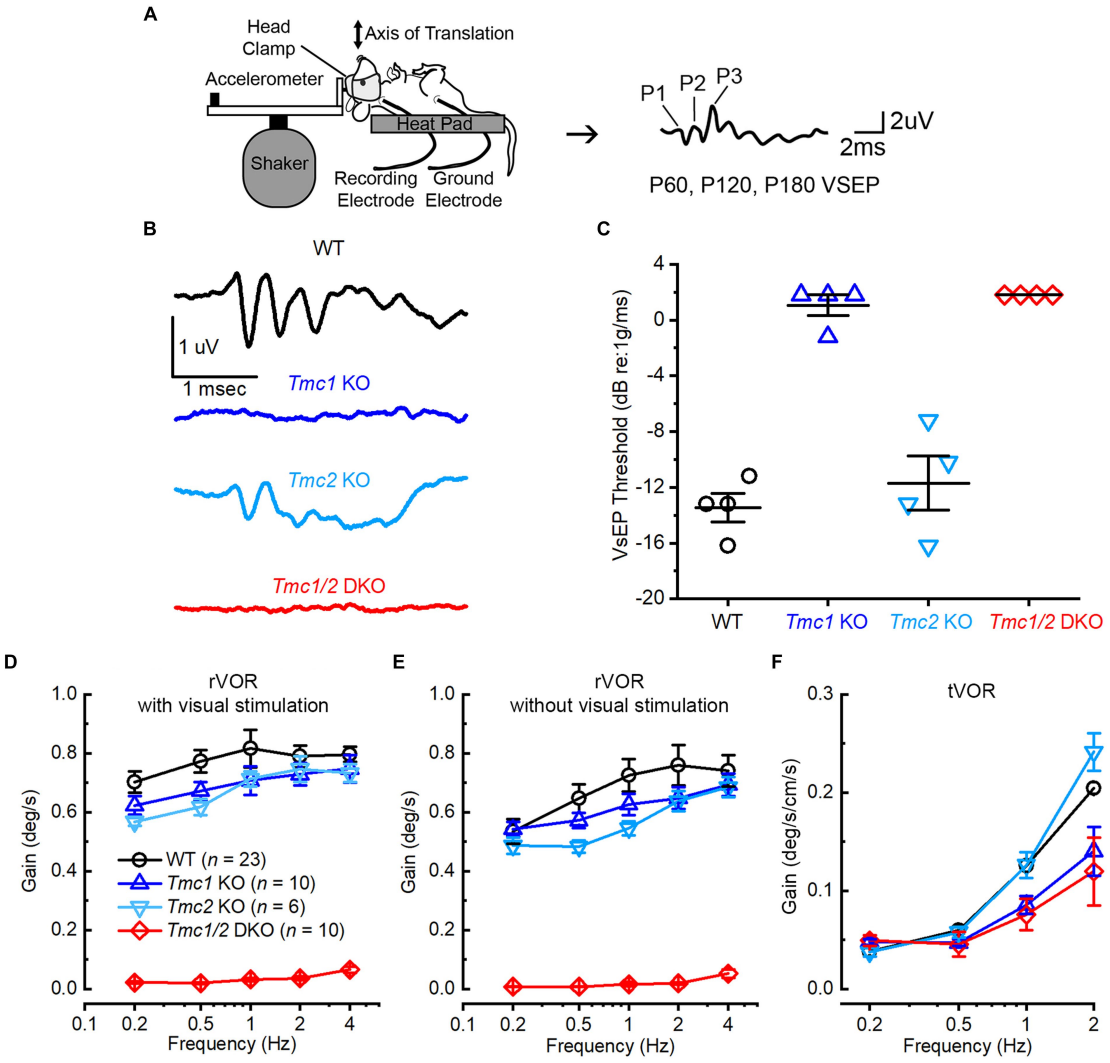
Phenotypic characterization of WT and *Tmc* mutant mice. **(A)** Schematic of VsEP stimulation and acquisition, modified from (30). **(B)** Representative VsEP waveforms of WT (black), *Tmc1* KO (blue), *Tmc2* KO (light blue), and *Tmc1*/*2* DKO (red) mice evoked by 1.6 dB re: 1 g/ms stimulation. Scale bars apply to all traces. **(C)** VsEP thresholds for individual mice (data points) and mean ± SEM values (bars). **(D,E)** Mean ± SEM rotational VOR (rVOR) response gains with and without visual stimulation in adult mice for each genotype with numbers mice tested indicated. **(F)** Mean ± SEM translational (tVOR) response gains in adult mice for each genotype.

*Tmc1* has been characterized as a necessary gene and protein for hearing and cochlear hair cell survival (5, 25), but less is known regarding its necessity for vestibular function. TMC1 and TMC2 proteins are localized to the tips of stereovilli in vestibular hair cells (29), and recordings from type II extrastriolar hair cells of *Tmc1* KO mouse utricles show differences in conductance and Ca^2+^ permeability relative to *Tmc2* KO and WT hair cells (6). To evaluate otolith function in *Tmc* mutant mice, we measured VsEPs (Figure 7A). Notably, *Tmc1* KO mice failed to produce VsEP waveforms during head translation events at even the highest stimulus intensity, and thus the mice lacked VsEP thresholds similar to *Tmc1*/*2* DKO mice (Figure 7B). *Tmc2* KO mice, on the other hand, produced characteristic VsEP waveforms with mean thresholds that were not significantly different from WT (WT: −14.50 ± 0.65 dB; *Tmc2* KO: −13.50 ± 1.94 dB, *n* = 4, *p* = 0.55) suggesting *Tmc2* is not required for linear VsEPs (Figure 7C).

Interestingly, both *Tmc1* KO and *Tmc2* KO vestibulo-ocular reflexes exhibited distinct phenotypic differences compared to WT mice. *Tmc1* KO mice exhibited decreases in both rotational VOR (rVOR) and translational (tVOR) gains (Figures 7D–F), which assay semicircular canal and otolith function, respectively. In contrast, *Tmc2* KO mice exhibited nearly the same tVOR gains as WT mice (Figure 7F). Similar to their failure to elicit VsEP signals, *Tmc1* KO and *Tmc1*/*2* DKO mice had significantly reduced tVOR gains relative to WT and *Tmc2* KO mice (Figure 7F). Taken together, the VsEP and VOR data suggest that *Tmc1* and *Tmc2* have distinct contributions to vestibular function; in particular, *Tmc1* contributes strongly to otolith organ function in adult mice.

### AAV-mediated replacement of *Tmc1* restores VsEP thresholds

Previously, our lab has shown *in vitro* adenoviral-mediated delivery of *Tmc1* transcripts restored mechanosensory transduction in type II utricle hair cells (5, 6). Additionally, we demonstrated that *Tmc2* gene replacement can restore VOR function in *Tmc1*/*2* DKO mice (7). However, no *in vivo* functional restoration experiments have been evaluated for *Tmc1* gene replacement in vestibular hair cells. Thus, we sought to introduce the *Tmc1* coding sequence into the vestibular system through left utricular injection of 1 μL of AAV9-PhP.B-*CMV*-*Tmc1ex1*-*WPRE* in P0-P1 mice. *Tmc1* KO mice in the negative control group were injected with 1 μL of AAV9-PhP.B-*CMV*-*eGFP*-*WPRE*. Previous work demonstrated that the injection technique and viral capsid efficiently and safely drive expression of GFP in utricle and saccule hair cells (18) and that AAV9-PhP.B-*CMV*-*Tmc1ex1*-*WPRE* restores auditory function in *Tmc1* KO mice (8, 26).

**Figure 8:**
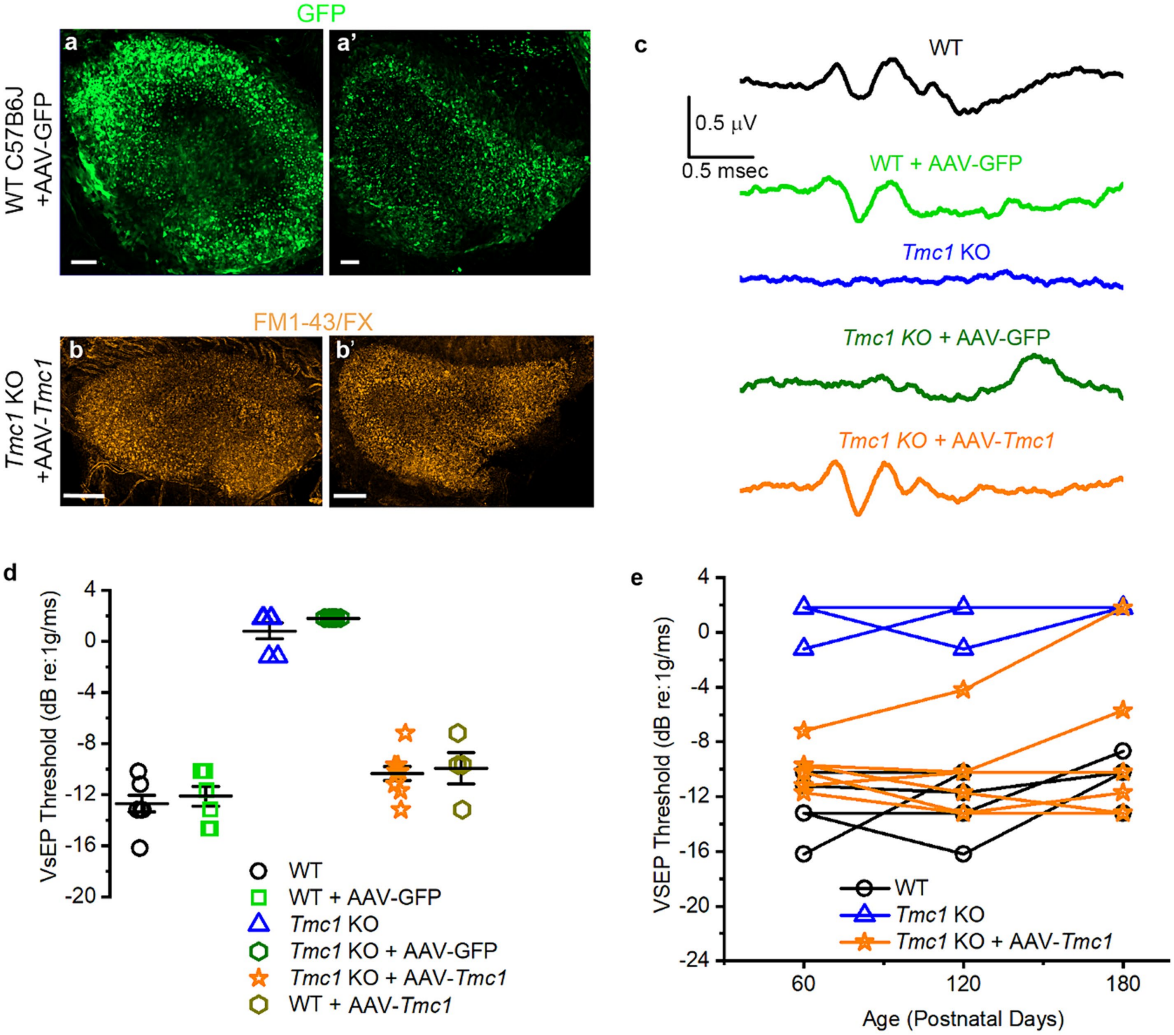
Recovery of VsEP thresholds after gene replacement with AAV-*Tmc1*. **(A)** Utricle injection of AAV-GFP in the utricle **(A)** and saccule **(A′)**. Scale bars = 50 μm. **(B)** FM1-43/FX uptake in the utricle **(B)** and saccule of a *Tmc1* KO mouse **(B′)** following AAV-*Tmc1* injection. Scale bars = 50 μm. **(C)** Representative VsEP traces evoked by −4.4 dB re: 1 g/ms stimulation and recorded from P60 mice for the genotypes and conditions indicated. Scale bars apply to all traces. **(D)** Mean ± SEM VsEP thresholds at P60 mice for the genotypes and conditions indicated. **(E)** VsEP thresholds from individual mice (WT, *Tmc1* KO and AAV-*Tmc1-*injected) recorded at P60, P120, and P180.

After neonatal injection and maturation of the vestibular system, we found robust GFP expression in both saccules and utricles of injected mice at P60 (Figure 8A). AAV9-PhP.B-*CMV*-*Tmc1ex1*-*WPRE* injection restored functional mechanosensory transduction in vestibular hair cells, as evidenced by FM1-43/FX uptake (Figure 8B), which was comparable to WT (Figures 3A–D). To test recovery of vestibular function we recorded VsEPs from injected WT and *Tmc1* KO mice (Figure 8C). Results showed that a single inner ear injection of AAV9-PhP.B-*CMV*-*Tmc1ex1*-*WPRE* was sufficient to restore VsEP peaks and thresholds in *Tmc1* KO mice, whereas uninjected mice or *Tmc1* KO mice injected with AAV9-PhP.B-*CMV*-*eGFP*-*WPRE* had no measurable VsEP responses (Figures 8C,D). WT mice injected with either AAV9-PhP.B-*CMV*-*eGFP*-*WPRE* or AAV9-PhP.B-*CMV*-*Tmc1ex1*-*WPRE* had no change in VsEP thresholds, indicating the injection technique and viral vectors had no deleterious consequences on vestibular function. To track the durability of the response, VsEPs were tested at P60, P120, and P180. VsEPs were not recorded later than P180 due to previous reports that VsEP thresholds are significantly elevated in C57B6 mice, which may not be representative of normal vestibular function (19). Tracking individual mouse thresholds over time showed that gene replacement of *Tmc1* through viral delivery effectively restored and maintained thresholds near WT levels for up to 6 months without need for a second treatment (Figure 8E).

### VsEP thresholds correlate with *Tmc1* transfection efficiency

**Figure 9:**
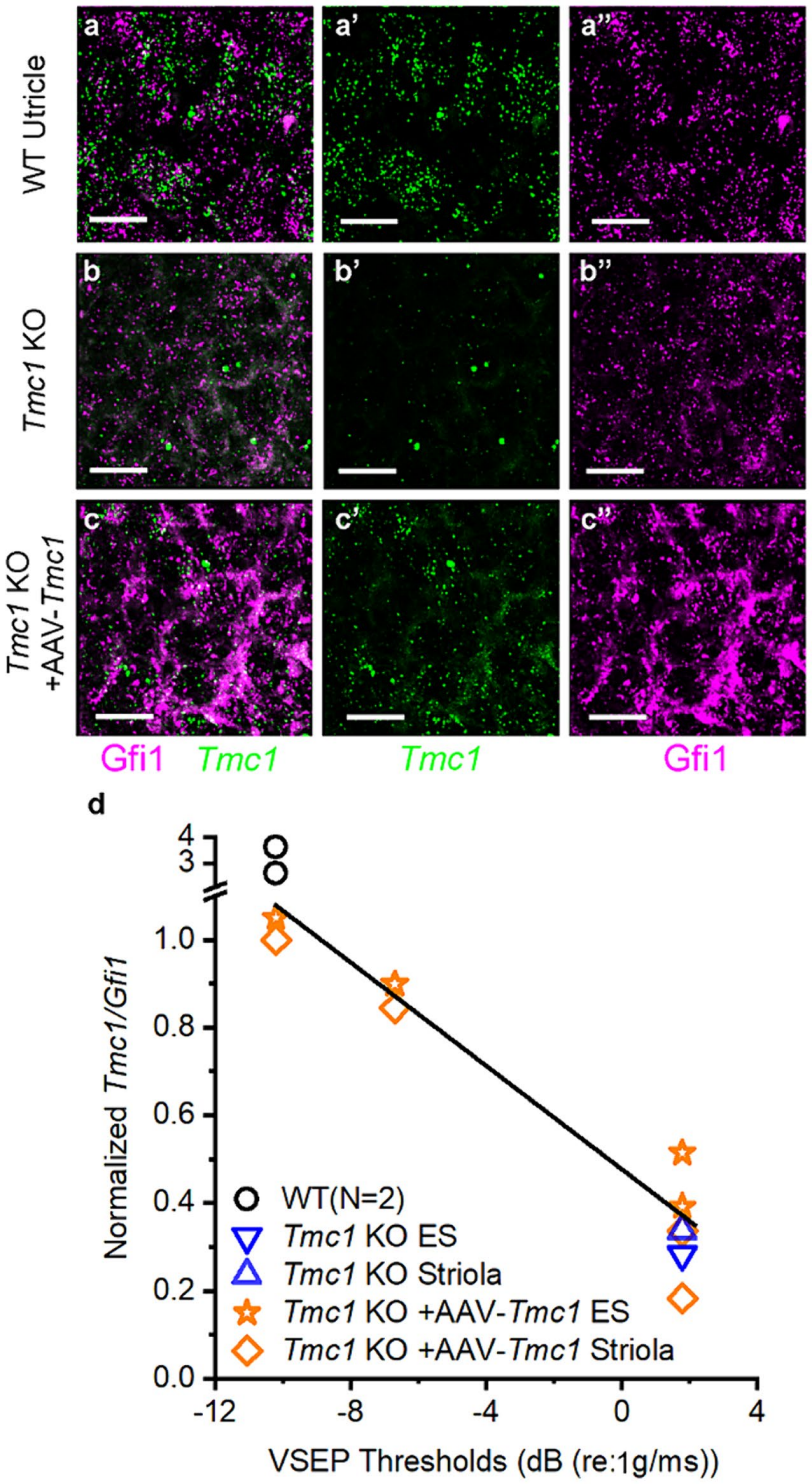
VsEP thresholds correlate with *Tmc1* transcript levels. **(A–A″)** Wholemount FISH-HCR of P60 WT mouse utricle, labeled with *Gfi1* and *Tmc1*. **(B–B″)** *Tmc1* KO and **(C–C″)** *Tmc1* KO mice injected at P1 with AAV-*CMV*-*Tmc1ex1*-*WPRE*. Mice with variable VsEP recoveries were also examined with FISH-HCR. Scale bars = 10 μm. **(D)** Quantification of *Tmc1* normalized to *Gfi1* from individual mice revealed higher expression level in mice with lower VsEP thresholds in both striolar and extrastriolar domains. Data were fitted with a linear regression with a slope of −0.059 and *R*^2^ = 0.93.

In principle, functional restoration of vestibular signaling is expected to correlate with the efficiency of gene delivery to target tissues. Accordingly, we hypothesized that the degree of VsEP threshold recovery in *Tmc1* KO mice would vary with *Tmc1* expression level following gene replacement therapy. To assess the efficacy of AAV delivery to vestibular hair cells, neonatal (P0-P1) *Tmc1* KO mice injected with AAV9-PhP.B-CMV-*Tmc1ex1*-*WPRE* were assessed for VsEP recovery at P60, and ears were collected for wholemount FISH HCR analysis (Figures 9A–C). *Tmc1* expression level was then plotted as a function of VsEP thresholds level to evaluate correlation between *Tmc1* mRNA and the level of functional vestibular recovery (*n* = 4 mice, 109 striolar and 111 extrastriolar cells). Results showed a linear relationship between VsEP thresholds in *Tmc1 KO* mice and the amount of normalized *Tmc1*/*Gfi1* in striolar hair cells (*R*^2^ = 0.97) (Figure 9D). Likewise, extrastriolar levels of normalized *Tmc1* correlated with improved recovery of VsEP thresholds (*R*^2^ = 0.97). No difference was observed between *Tmc1* expression in striolar versus extrastriolar regions within each mouse.

### Model of *Tmc1* dependence in the saccule

**Figure 10:**
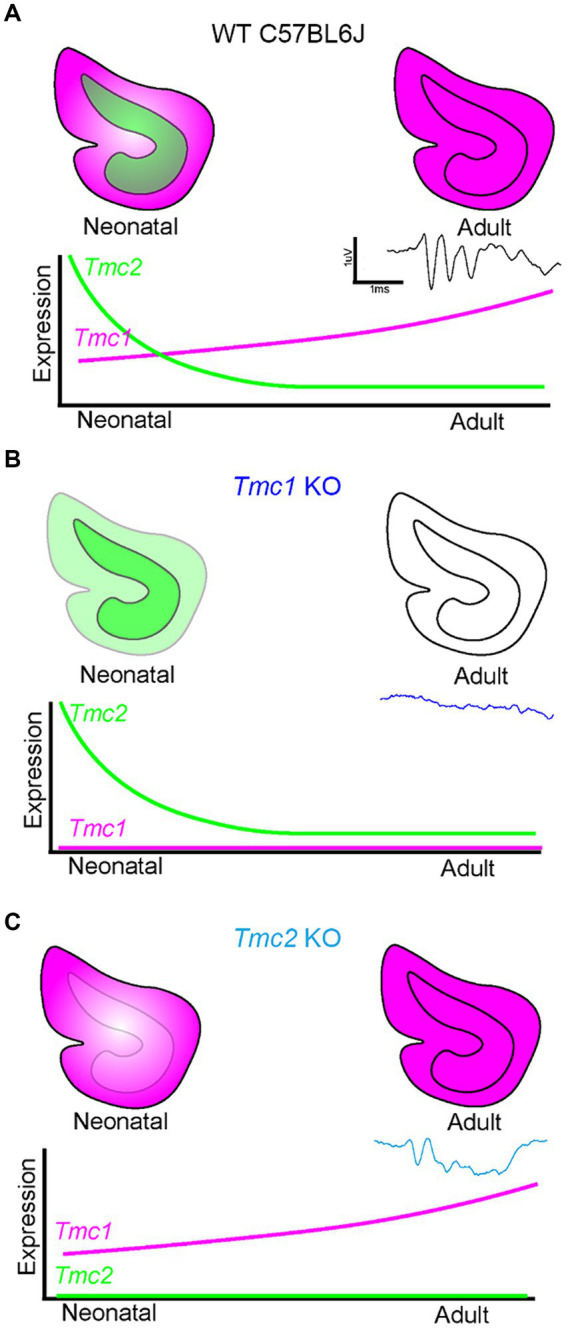
Model of *Tmc1-*dependent VsEP generation in the mouse saccule. **(A)** WT saccules express *Tmc2* (green) early during neonatal development, but it is no longer present in mature saccules. *Tmc1* expression (magenta) increases with maturity and is sufficient to maintain VsEPs in adulthood. **(B)** In *Tmc1* KO mice, *Tmc2* expression is not sufficient to maintain adult VsEP signals. **(C)** In *Tmc2* KO mice, remaining *Tmc1* expression is sufficient to maintain adult VsEP responses.

These data are consistent with a model in which transient striolar expression of *Tmc2* early in neonatal vestibular development at a time point when low-level *Tmc1* expression resides primarily in extrastriolar hair cells of the vestibular maculas (Figure 10A). Subsequently, qPCR analysis and FISH HCR imaging show that *Tmc2* declines as mouse maculas mature while *Tmc1* expression continues to increase and expand into the striolar domain. As a result, the saccule likely becomes more functionally dependent on *Tmc1* expression with age. This is supported by FM1-43 dye uptake efficiency diminishing in extrastriolar hair cells over time and the loss of responsiveness to lateral translational movement (tVOR). As a result, *Tmc1* KO mice lack sufficient levels of *Tmc2* to maintain VsEP responsiveness in adulthood (Figure 10B). In contrast, *Tmc2* KO vestibular organs fail to take up FM1-43 initially at P3, but by P60, *Tmc1* expression has both increased and expanded into the striolar domain, presumably compensating for the loss of *Tmc2* and enabling near WT VsEP and tVOR responses (Figure 10C).

## Discussion

Human *TMC1* is amongst a cadre of genes subject to pathogenic mutations associated with hearing loss (10) and, thus, an appropriate target for therapeutic intervention (26). Despite the prominence of hearing loss amongst *TMC1* patients, vestibular dysfunction has not been associated with *TMC1* mutations thus far. However, this may be in part due to the limited availability of robust vestibular diagnostic testing within audiology/otolaryngology clinics. Like human patients, *Tmc1* KO mice do not display observable vestibular deficits in terms of circling, head tossing, aberrant posture, or head tilt. As such, we wonder whether data from *Tmc1* KO mice, which lack VsEPs and normal tVORs, suggest a similar phenotype may be hidden within human patients harboring *TMC1* mutations.

Loss of VsEP in *Tmc1* KO mice is a surprising result, given recent evidence supporting the notion that striolar hair cells and corresponding afferents predominantly contribute to VsEP signal (31–33). Interestingly, *Tmc1* KO mice retain normal expression of calcium buffering proteins OCM and calretinin, canonical indicators of striolar type I hair cells and their complex calyceal afferents, respectively. *Tmc1* expression in striolar hair cells increases from P3 to P60 but is expressed at higher levels in extrastriolar hair cells at early stages, a finding consistent with recent RNAseq datasets in other vertebrates (12, 34). FM1-43 dye uptake in the striola appears to be unaffected by loss of *Tmc1*. Instead, FM1-43 uptake is reduced in the extrastriolar region of the utricle and saccule of *Tmc1* KO mice. These results could mean that extrastriolar hair cells contribute meaningfully to the VsEP signal. There is precedence for extrastriolar contribution to VsEP signal from experiments using *Emx2-Cre*; *Tmie* CKO mice where lateral hair cells lack mechanotransduction and show elevated VsEP thresholds for positive head movements (31). Interestingly, higher levels of *Tmc1* in extrastriolar hair cells may be influenced by the recent finding that it is upregulated by the lateral extrastriolar transcription factor *Emx2* (23). Extrastriolar *Tmc1* contributes to VsEP signal generation, and *Tmc1* gene replacement restores this function for up to 6 months.

Several reports suggest a slight elevation in the ratio of type I to type II hair cells in the striolar region of the mouse utricle compared to the extrastriolar region (35–39). Thus, we wonder whether differences in regional FM1-43 dye uptake may be a result of heterogeneity of *Tmc* expression in different vestibular hair cell types. Recently, it has been shown that *Tmc1* expression is largely absent from the striolar zone of the zebrafish utricle, and *Tmc2a* and *Tmc2b* confer different frequency sensitivities in vestibular hair cells (15). Similarly, loss of *Tmc1* in tall hair cells results in substantial reductions in mechanosensitivity, and hair cells that use *Tmc1* are found in the posterior region of the saccule (34).

Arguably, the lack of a striolar phenotype in *Tmc1* KO mice and the limited expression of *Tmc1* in the striola point to the opposite conclusion that the striola may not be as relevant for VsEP generation as previously thought. To this point, *Tmc2* KO mice lack striolar FM1-43 uptake despite retaining normal VsEP thresholds. *Tmc2* KO mice also progressively lose calretinin expression from striolar calyxes. Calretinin acts as a potent cytosolic buffer capable of sequestering free Ca^2+^ and has been previously shown to regulate synaptic efficacy in auditory synapses during high-rate activity (40, 41). As the more Ca^2+^ permeable of the two channels, loss of even low levels of *Tmc2* may affect post-synaptic afferent neurons, which contact type I striolar hair cells uniquely without affecting VsEPs. Interestingly, presynaptic ribbon distribution appears to be unaffected by loss of *Tmc2*. Another explanation for phenotypic differences between *Tmc1* KO and *Tmc2* KO may be from non-reciprocal striolar compensation: i.e., *Tmc2* cannot compensate for *Tmc1* loss in the striola, but *Tmc1* can compensate for loss of *Tmc2* in the striola.

Therapeutically, *Tmc2* may exhibit unique potential for gene replacement therapy due to previous observations of its ability to rescue *Tmc1*/*2* DKO more effectively than *Tmc1* in utricular hair cells *in vitro* (5, 6). *Tmc2* is also capable of *in vivo* rescue of vestibular dysfunction in *Tmc1*/*2* DKO mice (7). Thus far, *Tmc2* mutations have not been identified as a cause of hearing or balance problems in human patients, but *Tmc2* may be effective for gene replacement in the absence of *Tmc1*. The unique temporal downregulation of *Tmc2* and increase in *Tmc1* over time suggest that there may be an expanded therapeutic window for introduction of *Tmc* gene replacement therapy that may function differently at adult stages. While cochlear hair cells die early in *Tmc1* mutant mice, vestibular hair cells persist, which makes the potential therapeutic window an important area for future research.

Notably, in WT mice, both the loss of vestibular hair cells and elevation of VsEP thresholds can occur by 6 months of age, but this is often considered premature relative to age-related vestibular hair cell loss in mice (42). Additionally, age-related hair cell loss tends to be more severe in the semicircular canals of mice and humans than in the utricle and saccule (43–46). The hair cell loss and calretinin depletion phenotypes may be an effect of a higher endolymphatic potential (+9 mV) the saccule than in the semicircular canals (+3 mV) combined with a more severe ionic disequilibrium due to loss of *Tmc1* and *Tmc2* (47). Unfortunately, this does not provide an adequate explanation for why the utricle seems unaffected in both *Tmc2* KO and *Tmc1*/*2* DKO. The underlying differences in utricle versus saccule sensitivities to gene mutation and loss are another important area for further research and may inform future gene therapy approaches as well.

## Data availability statement

The raw data supporting the conclusions of this article will be made available by the authors, without undue reservation.

## Ethics statement

The animal study was approved by Boston Childrens Hospital IACUC. The study was conducted in accordance with the local legislation and institutional requirements.

## Author contributions

ER: Formal analysis, Investigation, Methodology, Writing – original draft, Writing – review & editing. JL: Formal analysis, Investigation, Writing – review & editing. MM: Formal analysis, Writing – review & editing. HZ: Formal analysis, Investigation, Writing – review & editing. WZ: Formal analysis, Investigation, Writing – review & editing. GG: Conceptualization, Formal analysis, Funding acquisition, Investigation, Supervision, Writing – review & editing. JH: Conceptualization, Data curation, Funding acquisition, Investigation, Methodology, Project administration, Supervision, Validation, Visualization, Writing – review & editing.
