# Supplementary material for: TMC function, dysfunction, and restoration in mouse vestibular organs
Source: Front Neurol. 2024 Apr 4;15:1356614. doi: 10.3389/fneur.2024.1356614 (PMC11024474; doi:10.3389/fneur.2024.1356614)
Supplement: Supplementary file 1 [file Data_Sheet_1.pdf]

Supplementary Material

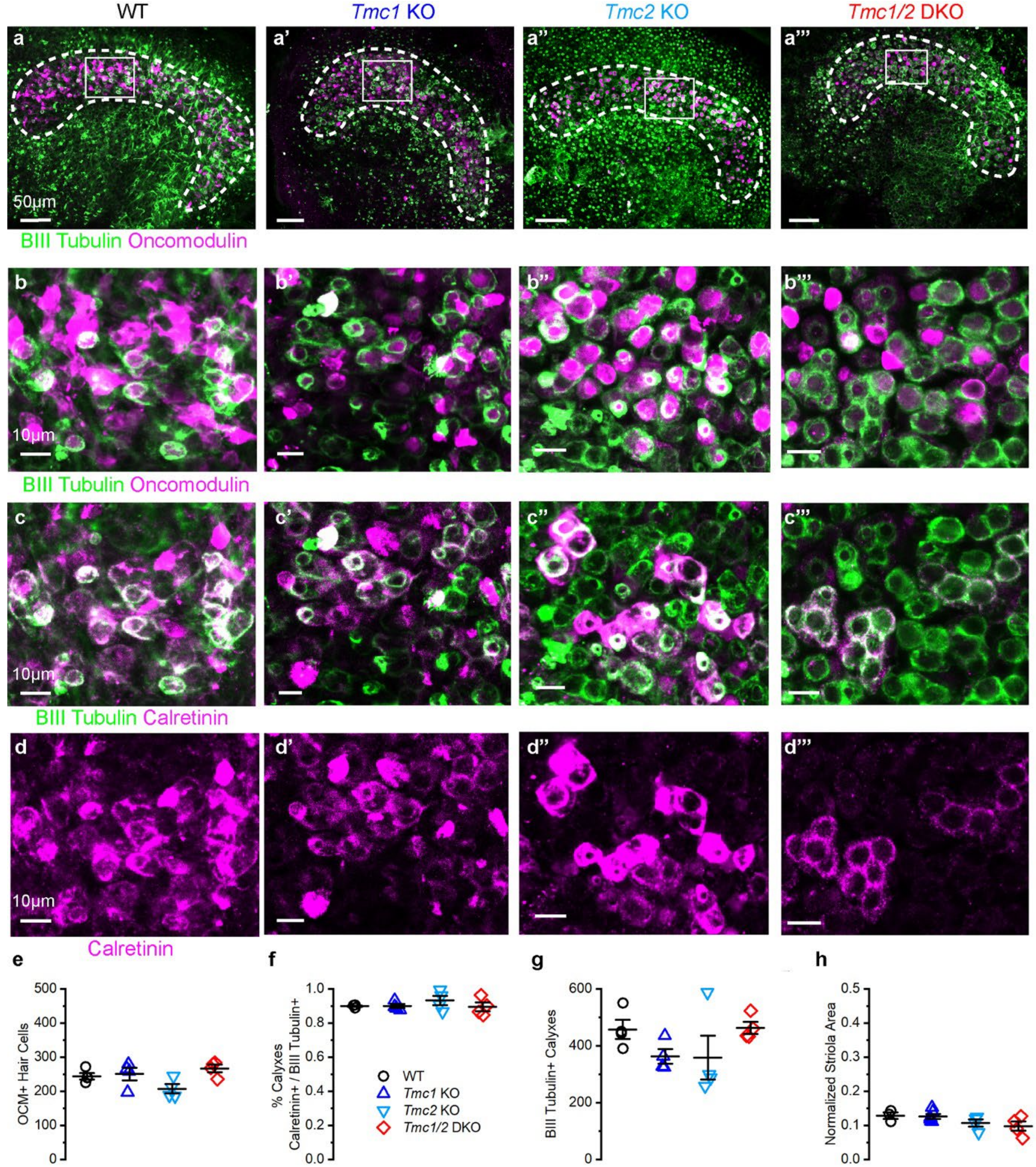

Figure S1: Immunostaining of P60 utricles from WT and *Tmc* mutant mice.

(a-a''') Utricular striola defined by OCM is present in all genotypes. Higher magnification, indicated by the box, is shown below. (b-b''') OCM labels type I hair cells in the striola. (c-c''') Calretinin expression labels striolar calyxes across all genotypes (magenta) and βIII tubulin expression labels calyxes across all genotypes (green). (d-d''') Calretinin signal labels complex calyxes (magenta). (e) Quantification of Oncomodulin+ (type I striolar hair cells) across genotypes at P60. (f) Quantification of the portion of striolar βIII tubulin(+) calyxes that also express calretinin across genotypes at P60. (g) Quantification of the total number of βIII tubulin calyxes in the striola across genotypes at P60. (h) Quantification of striolar area based on OCM signal normalized to the total sensory domain labeled by phalloidin. Scale bars = 50 µm for a, and 10 µm for b-d. Data points indicate values for each genotype with bars showing mean ± SEM in panels e - h.

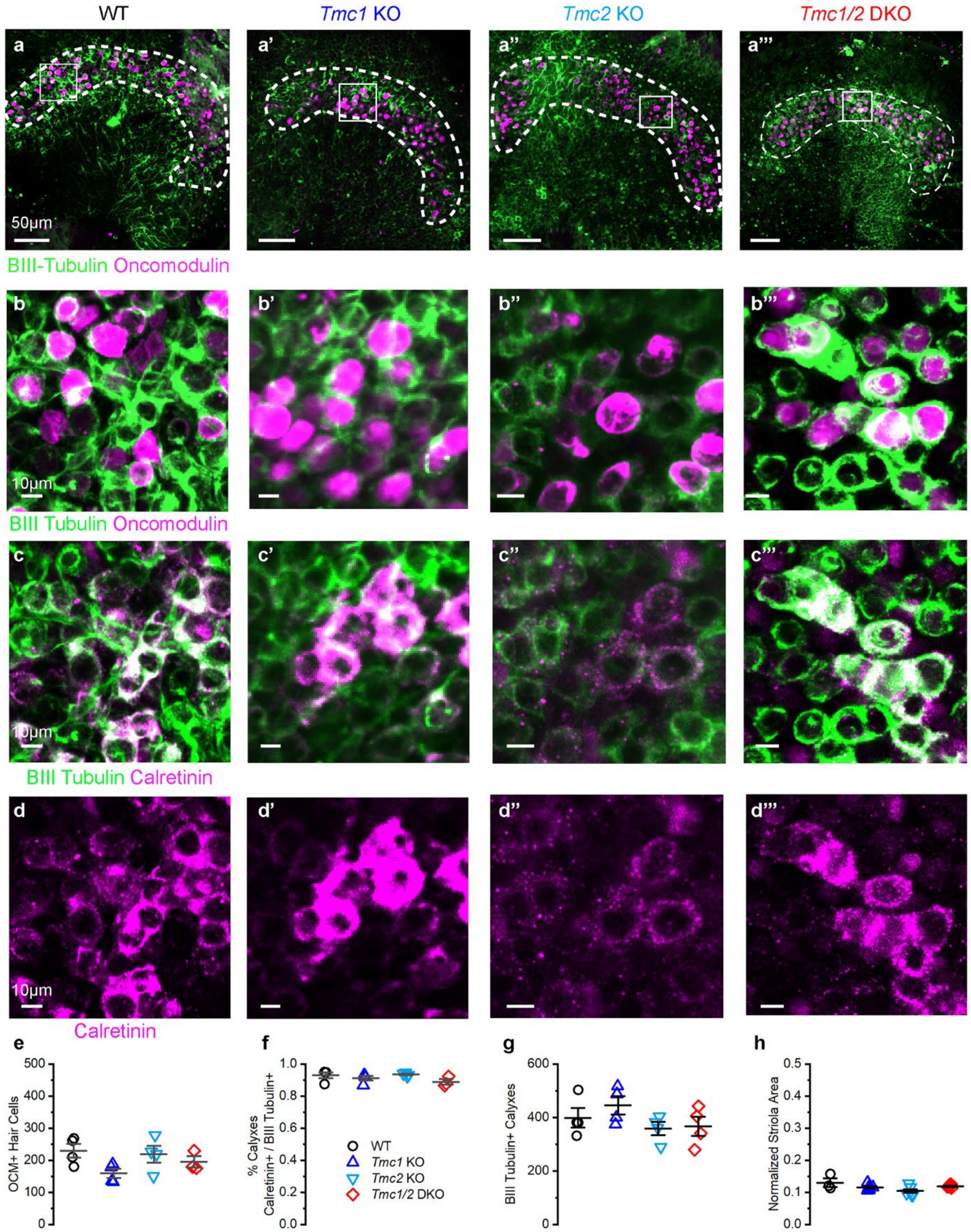

WT
Tmc1 KO
Tmc2 KO
Tmc1/2 DKO
a
a'
a''
a'''
50µm
BIII-Tubulin Oncomodulin
b
b'
b''
b'''
10µm
BIII Tubulin Oncomodulin
c
c'
c''
c'''
10µm
BIII Tubulin Calretinin
d
d'
d''
d'''
10µm
Calretinin
e
OCM+ Hair Cells
f
% Calyxes
Calretinin+ / BIII Tubulin+
WT
Tmc1 KO
Tmc2 KO
Tmc1/2 DKO
g
BIII Tubulin+ Calyxes
h
Normalized Striola Area

Figure S2: Immunostaining of P180 utricles from WT and *Tmc* mutant mice.

(a-a''') Utricular striola is defined by OCM labeling (dashed lines) and higher magnification indicated by square. (b-b''') OCM labels type I hair cells in the striola. (c-c''') Calretinin expression labels striolar calyxes across all genotypes (magenta) and βIII tubulin expression labels calyxes across all genotypes (green). (d-d''') Calretinin signal labels complex calyxes (magenta). (e) Quantification of the total type I striolar hair cell numbers across genotypes at P180. (f) Quantification of the portion of striolar βIII tubulin(+) calyxes that also express calretinin across genotypes at P180. (g) Quantification of total number of βIII tubulin calyxes in the striola across genotypes at P180. (h) Quantification of striolar area based on OCM signal normalized to the total sensory domain labeled by phalloidin. Scale bars = 50 μm for a, and 10 μm for b-d. Data points indicate values for each genotype with bars showing mean ± SEM in panels e - h.

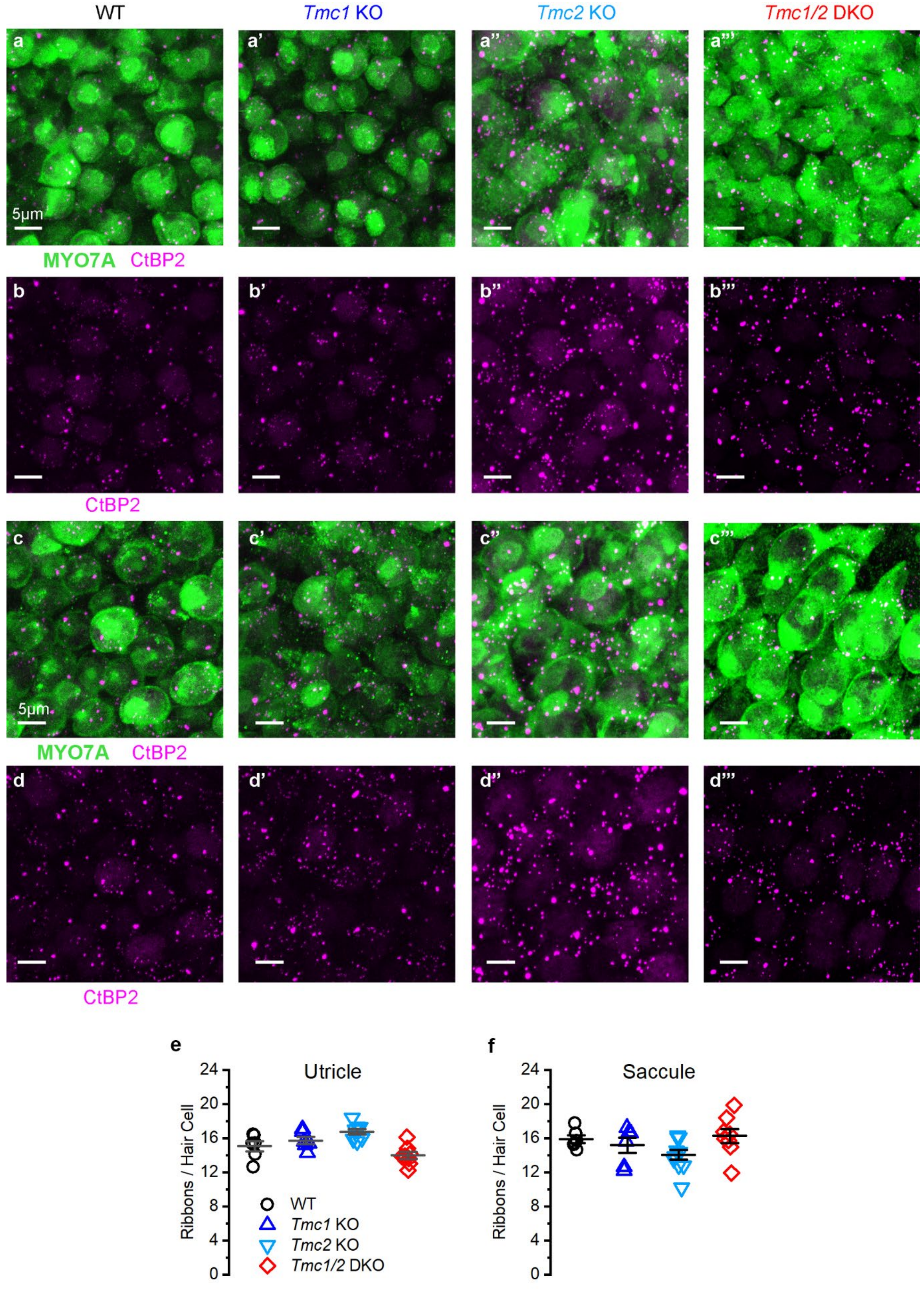
WT
Tmc1 KO
Tmc2 KO
Tmc1/2 DKO
a
a'
a''
a'''
5μm
MYO7A CtBP2
b
b'
b''
b'''
CtBP2
c
c'
c''
c'''
5μm
MYO7A CtBP2
d
d'
d''
d'''
CtBP2
e
Utricle
Ribbons / Hair Cell
24
20
16
12
8
4
0
WT
Tmc1 KO
Tmc2 KO
Tmc1/2 DKO
f
Saccule
Ribbons / Hair Cell
24
20
16
12
8
4
0

Figure S3: Evaluation of presynaptic ribbons per hair cell across genotypes.

(a-a''') Utricular hair cells labeled with Myo7a antibody and presynaptic ribbons with CtBP2. (b-b''') CtBP2 alone (c-c''') Saccular hair cells labeled with Myo7a antibody and presynaptic ribbons with CtBP2 and (d-d''') CtBP2 alone. (e-f) Quantification of ribbons per hair cell for utricles and saccules across genotypes. Scale bars = 50 μm. Data points indicate values for each genotype with bars showing mean ± SEM in panels e & f.
